# Deep learning based assessment of hemodynamics in the coarctation of the aorta: comparison of bidirectional recurrent and convolutional neural networks

**DOI:** 10.3389/fphys.2024.1288339

**Published:** 2024-02-21

**Authors:** Jakob Versnjak, Pavlo Yevtushenko, Titus Kuehne, Jan Bruening, Leonid Goubergrits

**Affiliations:** Institute of Computer-assisted Cardiovascular Medicine, Deutsches Herzzentrum der Charité, Berlin, Germany

**Keywords:** congenital heart disease, synthetic cohort, magnetic resonance imaging, computational fluid dynamics, pressure gradient, machine learning

## Abstract

The utilization of numerical methods, such as computational fluid dynamics (CFD), has been widely established for modeling patient-specific hemodynamics based on medical imaging data. Hemodynamics assessment plays a crucial role in treatment decisions for the coarctation of the aorta (CoA), a congenital heart disease, with the pressure drop (PD) being a crucial biomarker for CoA treatment decisions. However, implementing CFD methods in the clinical environment remains challenging due to their computational cost and the requirement for expert knowledge. This study proposes a deep learning approach to mitigate the computational need and produce fast results. Building upon a previous proof-of-concept study, we compared the effects of two different artificial neural network (ANN) architectures trained on data with different dimensionalities, both capable of predicting hemodynamic parameters in CoA patients: a one-dimensional bidirectional recurrent neural network (1D BRNN) and a three-dimensional convolutional neural network (3D CNN). The performance was evaluated by median point-wise root mean square error (RMSE) for pressures along the centerline in 18 test cases, which were not included in a training cohort. We found that the 3D CNN (median RMSE of 3.23 mmHg) outperforms the 1D BRNN (median RMSE of 4.25 mmHg). In contrast, the 1D BRNN is more precise in PD prediction, with a lower standard deviation of the error (±7.03 mmHg) compared to the 3D CNN (±8.91 mmHg). The differences between both ANNs are not statistically significant, suggesting that compressing the 3D aorta hemodynamics into a 1D centerline representation does not result in the loss of valuable information when training ANN models. Additionally, we evaluated the utility of the synthetic geometries of the aortas with CoA generated by using a statistical shape model (SSM), as well as the impact of aortic arch geometry (gothic arch shape) on the model’s training. The results show that incorporating a synthetic cohort obtained through the SSM of the clinical cohort does not significantly increase the model’s accuracy, indicating that the synthetic cohort generation might be oversimplified. Furthermore, our study reveals that selecting training cases based on aortic arch shape (gothic *versus* non-gothic) does not improve ANN performance for test cases sharing the same shape.

## 1 Introduction

In recent years, technological developments have enabled the integration of artificial intelligence (AI), including machine learning (ML), into clinical practice (Asselbergs and Fraser, 2021). This promising development holds the potential to transform various aspects of medicine, ultimately advancing the concept of precision medicine and thereby improving healthcare across many domains - from diagnosis to treatment decision and planning. One of the most advanced applications of AI in clinical practice is the field of diagnostic imaging (Yasaka et al., 2018; van Leeuwen et al., 2021). Additionally, AI has demonstrated considerable success in automating the diagnosis of electrocardiographic data (Siontis et al., 2021).

Furthermore, technological advancements during the last 2 decades have facilitated the use of image-based computational fluid dynamics (CFD) analysis within the field of cardiovascular medicine (Morris et al., 2016). This approach, known as image-based CFD modeling of patient-specific hemodynamics, allows the computation of flow parameters with notably higher spatial and temporal resolutions than achievable by any existing *in vivo* imaging technique (Canè et al., 2022). The outcomes of these simulations could finally be used for clinical support, particularly in cardiovascular surgical planning and diagnostics (Morris et al., 2016). However, despite having many benefits, image-based CFD remains sparsely used in routine clinical practice, with a few exceptions such as the calculation of the Fractional Flow Reserve by HeartFlow (Taylor et al., 2013). Several factors contribute to the limited clinical integration. Notably, CFD demands long computation times, substantial computational resources, and experienced engineers to set up simulations correctly. Unfortunately, these limitations make CFD less usable with current clinical workflows (Huberts et al., 2018).

Recently, ML has been proposed as a valuable tool to enhance CFD methods. The primary objective of employing ML in CFD is to optimize various aspects, including the acceleration of simulations, as seen in direct numerical simulations (Bar-Sinai et al., 2019), the improvement of turbulence models, and the development of reduced-order models (Duraisamy et al., 2019; Vinuesa and Brunton, 2022). Furthermore, ML can serve as a low-dimensional approach to replace CFD by using deep learning (Yevtushenko et al., 2022; Yevtushenko et al., 2023).

For instance, Ferdian et al. (2022) demonstrated that spatiotemporal wall shear stress (WSS) in the aorta can be estimated using a convolutional neural network (CNN) based on U-Net architecture. They accomplished this by using data of four-dimensional phase-contrast magnetic resonance imaging (4D PC MRI) assessing a three-dimensional velocity field with various image resolutions as input. The success of their approach leads to the question of whether a similar approach could be applied to predict other hemodynamic parameters, such as blood pressure.

The major aim of the presented study is to advance the use of artificial neural network (ANN) for treatment decision support, building upon recent work by Yevtushenko et al. (Yevtushenko et al., 2022), for the calculation of the pressure drop (gradient) in coarctation of the aorta (CoA) using the CNN as an alternative approach to the bidirectional recurrent neural network (BRNN). CoA, a congenital heart disease characterized by aortic narrowing (stenosis), causes a high pressure gradient that affects human circulation (Kenny and Hijazi, 2011; Brown et al., 2013). In addition to introducing a change in network architecture (CNN vs. BRNN), the low-dimensional representation of the aortic shape (one-dimensional (1D) scalar values along the centerline) was replaced with a high-dimensional approach (two-dimensional (2D) cross-sections along the centerline) to potentially improve the ANN performance by providing a more spatially accurate representation of aortic shape. Furthermore, the study explores several aspects of ANN training, including the use of real *versus* synthetic aortic shapes with CoA and the impact of different anatomical pathologies, such as gothic arch shapes.

## 2 Materials and methods

In this study, data for training and testing two different ML models were used from a database provided in a recently published study (Yevtushenko et al., 2022). These data were derived from image-based CFD simulations of real patients as well as simulations based on synthetically generated boundary conditions, as described in our earlier work (Thamsen et al., 2021). Subsequently, the data generation procedure is briefly summarized:

• The aortic geometry was manually reconstructed from 3D steady-state free-precession (SSFP) magnetic resonance images (MRI) of the thoracic aorta. Additional information regarding the MRI device, MRI acquisition sequence, and the segmentation procedure, including surface reconstruction for CFD simulations, is described in our previous work (Yevtushenko et al., 2022).
• In this study, real data were extracted from 106 patients with CoA before treatment, 37 patients (a sub-cohort of 106 CoA patients) after treatment, and 85 healthy subjects, forming a real cohort of 228 cases. This cohort was also used to construct an SSM, which allowed the generation of synthetic cases.
• A subset of 139 cases from the real cohort was used to train ANNs.
• For accurate flow boundary conditions, 4D PC MRI was used. This included obtaining the inlet velocity profile and peak systolic flow rates at the ascending and descending aorta for the cases where PC MRI data were available. Otherwise, flow boundary conditions were synthetically generated, as described previously (Yevtushenko et al., 2022).
• A synthetic cohort of 2968 cases was generated based on the statistical shape model (SSM) aiming to expand the training database upon the real cohort. This entailed generating both boundary conditions needed for CFD simulations: the geometry of the aorta and the inlet as well as outlet flow rates. The SSM approach used linear principal component analysis as described in more detail here (Thamsen et al., 2021; Yevtushenko et al., 2022).
• Hemodynamics of CoA cases, both real and synthetic, were calculated using the commercial CFD solver Siemens STAR-CCM+, version 13.02 (Siemens PLM Software, Plano, TX, United States). Simulations were performed only at the peak systolic state to reduce computational costs of CFD simulations and because only this state of a cardiac cycle is required for the treatment decision according to the clinical guidelines (Baumgartner et al., 2010).
• To evaluate the performance of the trained ANNs, 18 real cases were reserved for testing. Among these, 13 cases were CoA patients, whereas 5 represented individuals with a healthy aorta. These cases were excluded from the training and validation process, solely reserved for testing, and were also not used for the development of the synthetic cohort to mitigate data leakage.

This chapter is further subdivided into four subsections (2.1–2.4) describing data structure and architecture for both ANNs: 1D BRNN and 3D CNN. The subchapter after (2.5) describes 4 ML experiments performed within the frame of this study aiming to assess various aspects of ANN performance. The final subchapter (2.6) provides an overview of the statistical tests used to evaluate the significance of the results.

### 2.1 One-dimensional bidirectional recurrent neural network data structure

The development of an ANN usually starts with a definition of the ANN’s output parameters as well as their dimensions and resolutions. In our case, the major aim of ANN development is the prediction of hemodynamic biomarkers that characterize aortic flow. These biomarkers could support clinical decision-making and are typically computed using CFD. CFD primarily calculates pressure and velocity vector fields with high spatial resolution, which allows calculating derivatives, e.g., pressure drop or WSS, as well as integral parameters such as surface-averaged WSS or pressure drop between the inlet and outlet. However, high spatial resolution quantitative data (velocity and/or pressure fields) provided by CFD is not directly employed in clinical decision-making. Therefore, an ML approach presents an opportunity to develop a model capable of directly predicting integral and derived hemodynamic parameters, while significantly reducing computational cost, functioning as a form of reduced-order modeling.

The following hemodynamic parameters were chosen to be predicted by the initially proposed ANN using 1D BRNN architecture:

• relative static pressure, mmHg
• wall shear stress (WSS), Pa
• secondary flow degree (SFD), -
• specific kinetic energy (KE), mJ/kg
• average turbulence kinetic energy (average TKE), mJ/kg
• maximum turbulence kinetic energy (maximum TKE), mJ/kg
• average velocity magnitude over cross-section, m/s
• maximum velocity magnitude over cross-section, m/s

The selection of hemodynamic parameters to be predicted by the ANN was based on the following considerations:

1.Pressure drop, which is calculated from the relative static pressure curve, was selected because this is a major quantitative clinical biomarker (if the invasive catheter-based pressure measurements are performed) used by clinicians to decide whether to treat the CoA. All static pressure curves calculated by CFD or predicted by ANN were set at the inlet to the fixed static pressure value of 120 mmHg. This is done to enable a comparison between ANN and CFD since CFD is unable to calculate systemic blood pressure and calculates pressure curve course only with an inlet static pressure defined by a user.
2.Velocity magnitude, which is clinically measured with doppler echocardiography, was selected because usually used to assess pressure gradient by using the Bernoulli equation.
3.TKE was proposed because aortic flow is associated with turbulent flow states. Respectively, the ability of the ANN to predict turbulent parameters is indirectly associated with the ability to correctly predict pressure and velocity parameters.
4.WSS was selected because this is currently one of the major hemodynamic biomarkers used in cardiovascular research that is associated with various pathologies, such as atherosclerosis, thrombus formation, aneurysm development, and vessel dilatation.
5.Specific (volume normalized) KE and SFD, which is calculated at each cross-section as a ratio of the mean in-plane to the mean through-plane velocity magnitudes, were selected because these are hemodynamic parameters of interest since their increased values are associated with flow features, such as recirculations, helicity, and swirl. These flow features characterize abnormal hemodynamics, which are associated with pathologies (e.g., bicuspid aortic valve (Thamsen et al., 2021) or cardiovascular diseases (e.g., aortic valve stenosis (Nordmeyer et al., 2019)), and affect other hemodynamic parameters, such as WSS, pressure drop, turbulence, or maximal velocity magnitude.

**FIGURE 1:**
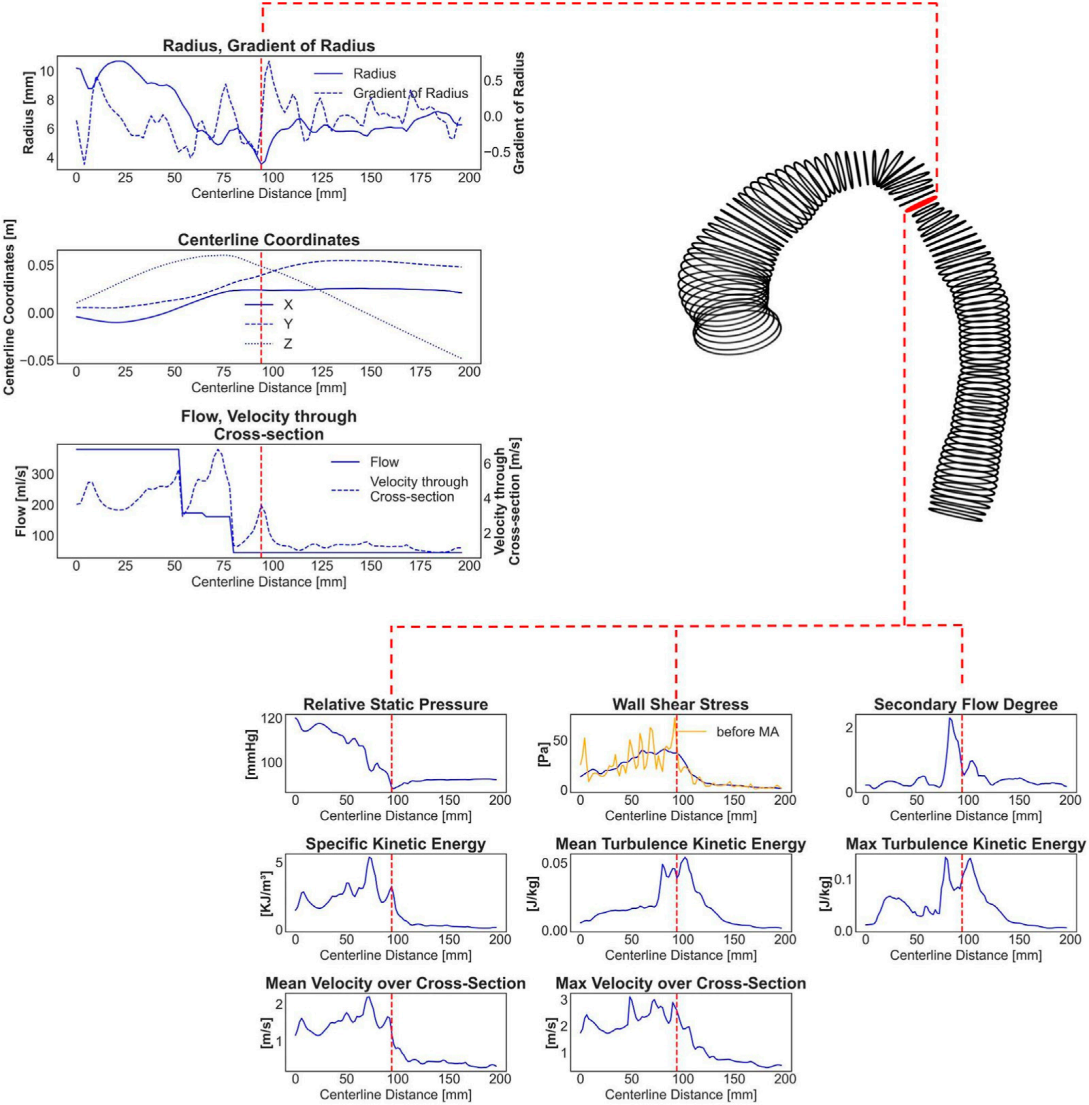
An exemplary real CoA case with a shape represented by circumferential lines. The red labeled line marks the stenosis site with the lowest diameter. Seven one-dimensional input parameters and eight centerline-aggregated output parameters, calculated by CFD and prepared for the ANN training, are shown. The yellow curve in the output WSS curve represents original WSS values with very high variance, whereas the blue curve shows 12-window averaged (smoothed) WSS data.

As part of our reduced-order modeling strategy, we proposed to assess hemodynamic parameters in a 1D centerline-aggregated format, instead of the high-resolution 3D CFD data. This transformation involved the following steps:

1.Generation of a discrete centerline: To start, a discrete centerline along the case-specific surface model of the aorta was generated, with points spaced 2 mm apart.
2.Vessel cross-section creation: At each centerline point, vessel cross-sections were generated, representing a slice of the aorta that is perpendicular to the centerline. The area of each cross-section was calculated.
3.Vessel surface segments: Vessel surface rings were created between neighboring cross-sections, which are essential for calculating surface-averaged WSS values in relation to the respective cross-section point. Additionally, a moving average filter with a window width of 12 neighboring rings was applied to WSS centerline-based data due to its higher variance (see Figure 1). As a result, the ANN was trained to predict the segment averaged WSS instead of the exact average value for every given centerline point.
4.Calculation of hemodynamics: On each centerline point, the hemodynamic parameters, including relative static pressure, WSS, SFD, KE, TKE, and velocity, were locally averaged across cross-sections. Moreover, the maximum cross-sectional values for TKE and velocity magnitude were determined.

The ANN was trained to map aorta geometry and blood flow input information to the eight aforementioned hemodynamic parameters, which resembles CFD simulations in a reduced form. The input information was composed of seven features:

• radius (n × 1)
• gradient of radius (n × 1)
• centerline point coordinates (n × 3)
• blood flow rate (n × 1)
• average velocity magnitude through cross-section (n × 1)

where n denotes the number of centerline points, which was set to 178, aligning with the number of centerline points in the longest aorta within the clinical and synthetic cohort. Note that most cases had fewer centerline points. Consequently, zero padding was applied to all the subsequent centerline points after the outlet.

The radius was derived from the maximum inner sphere that could fit into the aorta at each centerline point. The gradient of the radius was computed using second-order accurate central differences.

$$g_{x}=\frac{\;r_{x+1}-r_{x-1}}{2h},x\in \left[2,n\;-1\right]$$

$$g_{n}=\frac{\;r_{n}-r_{n-1}}{h}$$

$$g_{1}=\frac{\;r_{2}-r_{1}}{h}$$

where *r* denotes the radius, g represents the gradient of the radius, *h* signifies the spacing between centerline points, and *n* stands for the total number of centerline points. Blood flow at each centerline point was derived from the ascending inlet flow and outlet flow of the branching vessels at the aortic arch. The initial ascending inlet flow rate is diminished by the outlet flow after each bifurcation. Finally, the average velocity magnitude through each cross-section was computed by dividing the flow rate values by the area of the circular cross-section:

$$v_{x}=\frac{f_{x}}{\pi r^{2}},x\in \left[1,n\right]$$

where v stands for the velocity through the cross-section, *f* denotes the blood flow, and *n* represents the total number of centerline points. An example of input and output data for a patient with CoA can be found in Figure 1.

### 2.2 One-dimensional bidirectional recurrent neural network architecture

**FIGURE 2:**
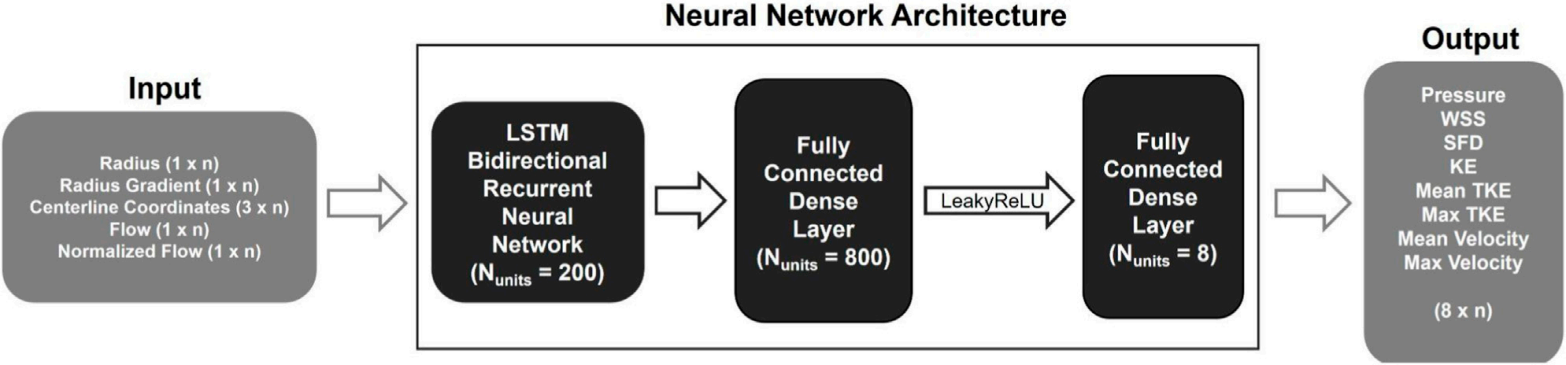
Schematic representation of the 1D BRNN architecture including input and output data with n representing the number of points describing the centerline.

The centerline-aggregated parameters of aortic flow can be considered as the duct flow of the blood in one direction. Hemodynamic values at any given centerline point are influenced by those preceding and following it; therefore, an ANN capable of capturing sequential dependencies is desirable. Hence, the recurrent neural network (RNN) was employed to predict hemodynamics along the centerline. The core ML approach was based on the model published earlier (Yevtushenko et al., 2022), and the implementation is done using TensorFlow 2.12.0 in Python (see Figure 2). It consisted of three major components:

• a long short-term memory (LSTM) BRNN,
• a densely connected neural network layer (dense layer) with Leaky rectified linear unit (ReLU) activation function, and
• an additional dense layer for hemodynamic outputs.

Leaky ReLU addresses the so-called dying ReLU problem, a limitation observed in the traditional ReLU activation function. Instead of assigning a gradient of 0 to all negative input values, Leaky ReLU introduces an extremely small linear component for negative inputs (Xu et al., 2015). The BRNN consists of two RNNs, one trained on the original input sequence, and the other on a reversed sequence. The hidden states of both RNNs are merged; in our case, the forward and backward outputs were concatenated, resulting in double the number of outputs that were fed into the next layer. The Leaky ReLU activation function with a slope of 0.3 for negative values was used in the following dense layer. Finally, another dense layer is added to map the outputs from the previous layer to hemodynamic values, resulting in an output space with a dimensionality equal to the number of output features.

We named this model 1D BRNN, highlighting the dimensionality of the training data and the core ANN. The 1D BRNN was trained with an initial learning rate of 0.001 and a batch size of 50. The learning rate was exponentially decreasing with the number of epochs. To optimize hyperparameters, 10-fold cross-validation was performed for each hyperparameter separately, with the optimal values found for each one indicated in brackets:

• scaling input and output data (no scaling)
• loss function (masked root mean square error (RMSE))
• optimizer (Adam)

Adam optimization is a stochastic gradient descent method based on adaptive estimation of first-order and second-order moments (Kingma and Ba, 2014). The masked RMSE considers only data within the aorta (non-zero output data) to compute the error.

It is worth noting that conducting a grid search to explore all possible hyperparameter combinations could have resulted in a different set of optimal hyperparameters. Due to its time-consuming nature, this approach was mostly avoided in this research. A coarse grid search was, however, used to determine the best combination of units (output dimensionality) for LSTM cells and the dense layer following the BRNN. The resulting 200 and 800 units, respectively, were found to provide the best performance. Once the optimal hyperparameters had been selected, the final model was trained on the entire training dataset. To prevent overfitting, a small validation dataset was still retained, and early stopping was used. Specifically, if the validation loss did not improve for 20 epochs in a row, the training process was stopped.

One important issue to consider is the potential for data leakage from the training to the validation datasets, as the synthetic cohort was generated from the SSM of the real cohort. However, if the estimation of general performance is equally biased for all validation datasets within cross-validation experiments, data leakage may not be a significant concern. After all, the main objective is to identify the best hyperparameters for the final model, which can also be based on relative performance. The held-out test was then used for the least biased estimation of general performance.

### 2.3 Three-dimensional convolutional neural network data structure

The centerline-aggregated method, which involves averaging cross-sectional surface values of hemodynamic parameters along the aorta centerline, as in the 1D BRNN approach, could result in a loss of potentially valuable information and, consequently, worse ANN performance. By retaining geometric cross-sectional surface information and employing a different ML approach a more accurate hemodynamic predictor could potentially be achieved. In this alternative approach, the cross-sectional surface values were stacked into a 3D array instead of using 1D values along the centerline.

To prepare the input and output data for training, 80 cross-sectional planes were extracted along the aorta centerline. Each cross-sectional plane had an initial resolution of 100 × 100, which was further decimated to 48 × 48. The decimation process involved applying a low-pass filter, specifically a Gaussian filter, to smooth the image before downsampling. This step helps to prevent aliasing artifacts. The standard deviation ( σ ) of the 2D Gaussian kernel was determined based on the following equation:

$$\sigma =\frac{s-1}{2}$$

where s is a ratio between the input dimension and the desired downsampled dimension. In our case, $s=\frac{\left[100,100\right]}{\left[48,48\right]},$ which results in $\sigma ≅\left[0.54,0.54\right]$ .

The spacing between the planes was set to 4 mm, twice the spacing used for training the 1D BRNN model. Note that not all 80 cross-sectional planes were needed for each case, as the aorta length varies from case to case. For all planes outside of the aorta, all input and output values were set to zero (zero padding).

**FIGURE 3:**
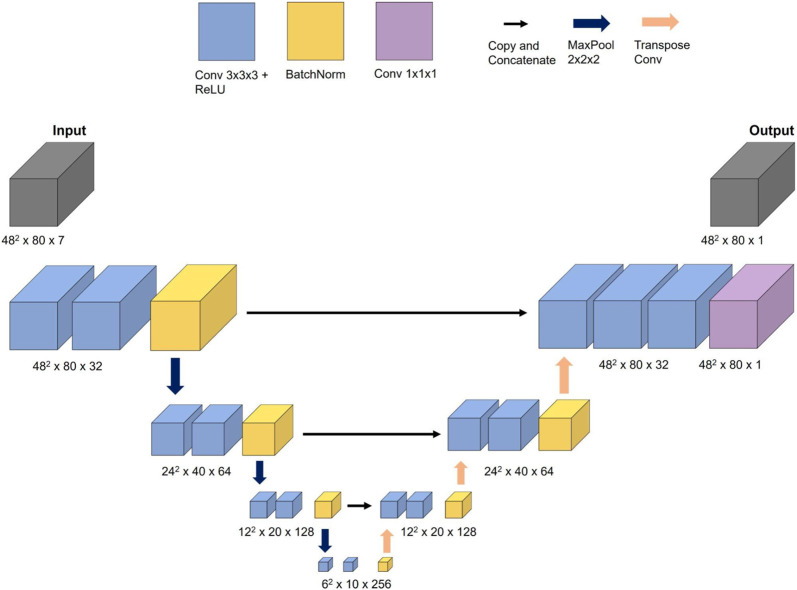
Schematic representation of the 3D CNN architecture including input and output data.

**FIGURE 4:**
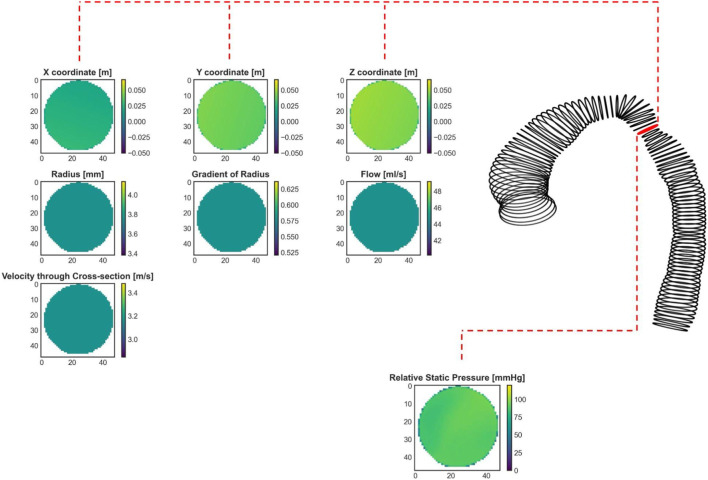
An exemplary real CoA case (same as in Figure 1) with shape represented by cross-sections. The red labeled cross-section marks the stenosis site with the lowest diameter. Seven input cross-sections and one output parameter, calculated by CFD and prepared for the ANN training, are shown.

The maximum length of the aorta that the 3D ML model could handle is 320 mm (80 cross-sections × 4 mm), which does not align with the maximum length used for 1D BRNN, which was 356 mm (178 centerline points × 2 mm). The reason behind this decision lies in the design of the U-Net model (see Figure 3), which requires dimensionalities that can be divided by 2 at least three times (three decoder and encoder layers) to ensure the same input and output dimensions. The input data consisted of 7 same features used for training the 1D BRNN:

• radius (48 × 48 × n × 1).
• gradient of radius (48 × 48 × n × 1).
• cross-sectional plane grid coordinates (48 × 48 × n × 3).
• blood flow (48 × 48 × n × 1).
• velocity magnitude through cross-section (48 × 48 × n × 1).

where n is equal to the number of cross-sectional planes, which was 80. The output data consisted of only one feature, which was blood pressure (see Figure 4). Only one feature was selected due to GPU RAM limitations to ensure a reasonably high batch size. Static pressure was chosen because it is the most predictive hemodynamic parameter for diagnosing patients with CoA.

To summarize, the training data consisted of an input data array of size (48, 48, 80, 7) and an output data array of size (48, 48, 80, 1). The first and second dimensions represent the height and width of each cross-sectional plane, respectively, the third dimension represents the number of cross-sectional planes, and the last represents the number of input or output features. Note that only the cross-sectional plane grid coordinates and pressure contain 3D information, whereas the remaining input features, including radius, gradient of radius, blood flow, and velocity through cross-sections, are scalar values. To accommodate this 3D data requirement, one approach is to assign constant values to the entire cross-sectional plane to represent scalar features (see the second and third rows of Figure 4). Our goal was to investigate whether including 3D geometric information provides additional insight for improving pressure course and pressure drop (gradient) prediction.

### 2.4 Three-dimensional convolutional neural network model architecture

For the task of predicting the spatial distribution of blood static pressure, a CNN inspired by the WSSNet (Ferdian et al., 2022) model was selected. The authors were able to predict WSS in the aorta from velocity sheets and coordinate flat maps using a U-Net-shaped CNN architecture. Given the similarities between the task of predicting WSS and blood pressure, the WSSNet was adapted for the current task.

The CNN network, referred to as 3D CNN in this study, consisted of three encoder and decoder blocks, with each block comprising two convolutional layers that used a 3 × 3 × 3 filter size and ReLU activation function. Batch normalization was applied at the end of each block. The encoder blocks used max pooling with a step size of 2 × 2 × 2, while the decoder blocks used transpose convolution instead of bilinear upsampling. One difference between these two methods is that the filter weights in bilinear upsampling remain constant during training, whereas in transpose convolution, they belong to trainable parameters. The filter size of 2 × 2 × 2 with a step size of 2 × 2 × 2 was set to avoid overlapping.

The 3D CNN was trained on an NVIDIA GeForce RTX 3090 Ti with 24 GB of memory. The initial learning rate was set to 0.0001 and the Adam optimizer with exponential annealing learning rate was used to find optimal weights. The model performance was evaluated using the masked RMSE loss function which ignores all values outside the aorta. A batch size of 8 was utilized for training. While the output was scaled by standard deviation, the input features were left unscaled because training the model with both scaled input and output features resulted in less accurate results. The measurement units of features can be seen in Figure 4. Note that the training of the 3D CNN takes considerably longer compared to the 1D BRNN, primarily due to the former’s higher number of trainable parameters (5,600,000) compared to the latter (660,000).

### 2.5 Artificial neural network performance analysis

Four ML experiments with different training data and ANN architectures were performed to analyze their impact on ANN performance:

1. 1D BRNN performance analysis.
2. Impact of a synthetic cohort on ANN training: real vs. synthetic.
3. Impact of an aortic arch shape on ANN training: gothic vs. non-gothic vs. mixed.
4. Impact of architecture on ANN training: 1D BRNN vs. 3D CNN.

#### 2.5.1 1D BRNN

To evaluate the performance of the 1D BRNN, the following scalar parameters were selected:

• Inlet-outlet pressure drop (PD),
• maximum wall shear stress (WSS_max_), and
• maximum velocity magnitude at the stenosis region (V_max_).

PD is equal to the difference between the inlet and outlet pressure. These parameters are of particular interest in patients with CoA, as they are known to exhibit high pressure drop, elevated WSS, and abnormal velocity profiles near the narrowed section of the aorta.

The Bland-Altman plots were used to assess the degree of agreement between the parameters obtained from the reference method (CFD) and the predictions from ML models. Various 1D BRNN models with optimal hyperparameters and random weight initialization were trained. Among these models, the one with the lowest validation loss was selected for the final analysis.

#### 2.5.2 Real *versus* synthetic training cohorts

To investigate the potential impact of training solely on real clinical or synthetic cases, two separate models were trained. The first model utilized 139 cases available from the clinical cohort, while the second model was trained on 139 cases, randomly selected from the larger synthetic cohort. The purpose of selecting a smaller training subset from the synthetic cohort, equal to the size of the clinical cohort, is to avoid bias that might arise if one model performs better simply because it is trained with more data. Both models were trained using the same optimal hyperparameters identified in the section on 1D ML model architecture (see section 2.2), changing only the batch size from 50 to 10 to increase the number of training iterations per epoch. It is generally considered advantageous if each epoch consists of multiple training iterations, as the weights go through the tuning phase more frequently. The dataset was further split into training and validation sets, with 116 cases used for training and the remaining 23 cases for validation. The training process was repeated five times, among which the one with the best performance, determined by the lowest validation loss, was selected for further analysis. Subsequently, the Bland-Altman plots of both models are compared.

#### 2.5.3 Gothic *versu*s non-gothic *versus* mixed cohorts

To explore the effect of anatomical pathologies, particularly the gothic aortic arch (Ou et al., 2004), yet another experiment was conducted. Both pathological shapes, CoA and the gothic aortic arch, are associated with a pathologically high pressure drop. However, flow phenomena causing these pressure drops are different. The pathology of the CoA is associated with an increased pressure drop in the aorta due to stenosis. Stenosis (vessel narrowing) is a type of the so-called form resistance. Flow separates downstream of the narrowing forming a recirculation zone, which is associated with energy loss (pressure drop). The gothic aortic arch, on the other hand, occurs when the width of the aorta (the distance between the ascending and descending aorta) becomes narrow, and the height of the arch is not maintained (Seo et al., 2015). The aortic arch, being a curved duct, represents another kind of form (shape) resistance. Curved vessels, especially those with high curvatures, also cause flow separation due to centrifugal force, resulting in pressure drop. However, the pressure drop due to vessel curvature in non-gothic shapes is usually negligible (Goubergrits et al., 2015; Bouaou et al., 2019). An ANN trained to predict the pressure drop in aortas without a gothic shape is not necessarily able to predict the pressure drop caused by a gothic aortic arch. Thus, the experiment aims to investigate whether there is a difference in performance when the 1D BRNN is trained only on cases with a gothic aortic arch. Three different models were trained using the following training data from the synthetic cohort:

• 227 synthetic cases with gothic-shaped aortas (gothic cases),
• 259 synthetic cases with non-gothic-shaped aortas (non-gothic cases),
• 112 synthetic gothic cases and 130 synthetic non-gothic cases (mixed cases).

Only synthetic cases were used for training the 1D BRNN model for this experiment because only a small fraction (about 20) of 139 cases of a real clinical cohort can be considered as cases with a gothic-shaped aorta. To examine and compare the performance of these models, a new held-out test dataset was created. The original test dataset had an imbalanced distribution with only 1 gothic case and 17 non-gothic cases. To address this, a new test dataset was formed with an equal number of gothic and non-gothic cases. Specifically, 15 gothic and 15 non-gothic cases from the clinical cohort were selected for testing. Note that these cases were previously used in the creation of the synthetic cohort, which introduces the possibility of data leakage from the training data to the testing data.

Similar to the previous experiment, each experiment was repeated five times with the same hyperparameters. The only difference is the batch size, which is set to 20. The dataset was further split into training and validation data with a ratio of 80/20. After five training sessions, the model with the lowest validation loss was selected for further performance assessment on testing data. The Bland-Altman plots of all three models were compared. The impact of aortic arch shape on ANN was investigated only for the PD parameter since the pressure drop is the main factor that can be affected by an aortic arch shape.

#### 2.5.4 1D BRNN *versus* 3D CNN

To assess the influence of architecture and training data dimensionality on the performance of the ANN, the 3D CNN described in Section 2.4 was trained. In order to compare the 1D BRNN and 3D CNN, Bland-Altman plots for PD were plotted. The cross-sectional pressure values (3D CNN predictions) were averaged along the centerline of the aorta to align the output dimensionality to 1D. This allows for a comparison of both model outputs. Furthermore, since PD is a scalar value, it does not provide information about the pressure profile along the aorta centerline. To assess the agreement of the pressure curves, the RMSE was calculated:

$$RMSE=\sqrt{\frac{1}{n}\sum_{i=1}^{n}{\left(p_{i}-{\hat{p}}_{i}\right)}^{2}}$$

where n denotes the number of centerline points, $p_{i}$ the pressure prediction by the 3D CNN or 1D BRNN on the *i*th centerline point, and ${\hat{p}}_{i}$ represents the CFD ground truth pressure value on the *i*th centerline point.

### 2.6 Statistical analysis

Statistical analysis was conducted using IBM SPSS Statistics software, version 28 (IBM, United States). For normally distributed parameters mean and standard deviation were reported, and normality of distribution was assessed using a Shapiro-Wilk test. Non-normally distributed parameters were described using median and interquartile range [IQR]. A paired two-tailed Student’s t-test was used to test for significant differences within normally distributed parameter differences, whereas Wilcoxon signed-rank tests were used for testing non-normally distributed parameter differences. All tests used a standard significance level of 0.05.

## 3 Results

### 3.1 1D bidirectional recurrent neural network performance

**FIGURE 5:**
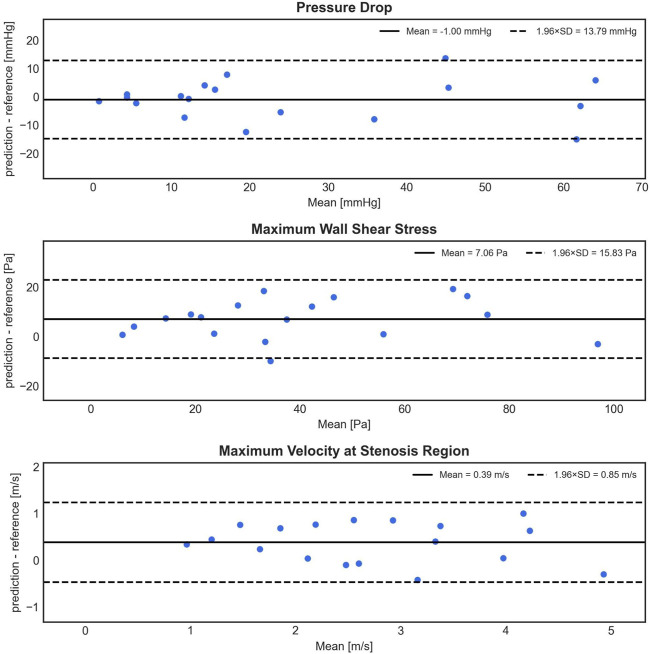
Bland-Altman plots depicting the difference (*y*-axis) between the predicted (1D BRNN) and reference (CFD) values against the mean of these values (*x*-axis) for PD, WSS_max_, and V_max_. The plots are based on 18 test cases that were not part of the training set and were also not used in creating the synthetic cohort.

In Figure 5, the Bland-Altman analysis of the mean prediction error for PD shows a small, non-significant underestimation of −1.00 ± 7.04 mmHg by 1D BRNN for 18 test cases (BRNN: 16.52 with [7.17–48.15] mmHg vs. CFD: 14.79 with [9.96–40.79] mmHg, paired Wilcoxon test, *p* = 0.616). On the other hand, the model tends to significantly overestimate WSS_max_, with an average error of 7.06 ± 8.08 Pa (43.38 ± 26.14 Pa vs. 36.31 ± 25.05 Pa, paired Student’s t-test, *p* = 0.002). However, it is worth noting that both WSS_max_ values are significantly higher than physiologic values of a few (<10) Pa in a healthy aorta (Callaghan and Grieve, 2018). Regarding the velocity, the V_max_ error also indicates a significant overestimation of 0.39 ± 0.43 m/s (2.93 ± 1.11 m/s vs. 2.54 ± 1.17 m/s, paired Student’s t-test, *p* = 0.002). However, this difference means according to the Bernoulli equation (PD = 4×V_max_^2^) an approximate PD of 0.6 mmHg, which is clinically negligible. Furthermore, the plots reveal a slight negative trend for V_max_, meaning that as the magnitude of velocity increases, the errors tend to shift toward the lower part of the interval.

**FIGURE 6:**
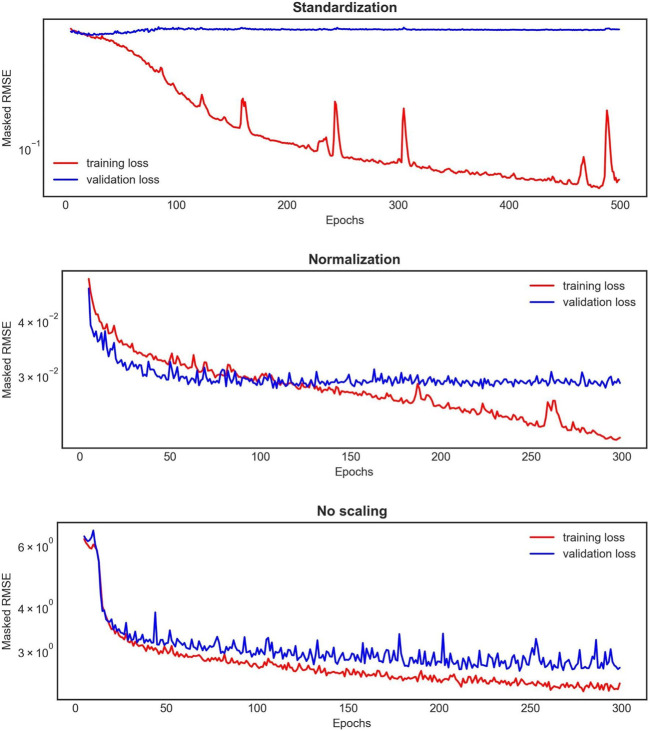
An example of the learning curves for one 1D BRNN training experiment out of the 10 conducted for each scaling method applied on both input and output features. The red curve represents the training loss, whereas the blue one represents the validation loss.

The 1D BRNN performance analyzed here is based on the model trained with non-scaled data. Throughout the model development process, various scaling methods were tested for both input and output data, revealing significant differences in training and validation loss curves (Figure 6). Notably, when the data were scaled, either standardized or normalized, the 1D BRNN model showed signs of overfitting. This is evident in Figure 6, where the training loss continues to decrease, while the validation loss either converges (in the case of normalization) or even increases (in case of standardization). Consequently, the final model was trained with non-scaled data.

### 3.2 Impact of a synthetic cohort on ANN training: real vs. synthetic cohorts

**FIGURE 7:**
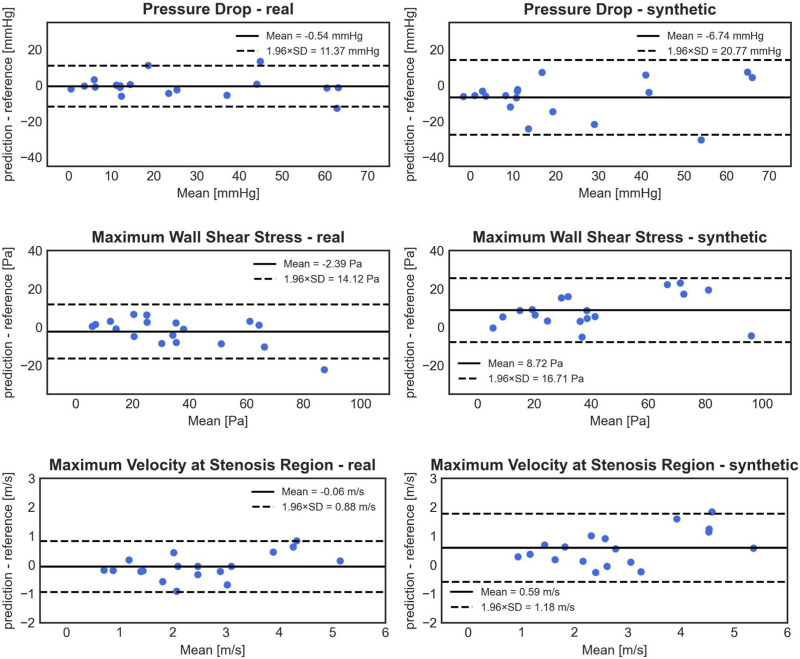
Bland-Altman plots comparing the accuracy of PD (1st row), WSS_max_ (2nd row), and V_max_ (3rd row) 1D BRNN predictions on test cases for the following experiments: trained only on real cases (left) vs. training only on synthetic cases (right).

The model trained on clinical (real) cases outperformed the one trained on synthetic cases, as observed in the Bland-Altman plots (Figure 7). This is evident from the lower standard deviation of the errors for PD (5.80 mmHg compared to 10.60 mmHg), WSS_max_ (7.20 Pa compared to 8.53 Pa), and V_max_ (0.45 m/s compared to 0.60 m/s). Additionally, the model trained on real cases exhibited less bias for all three parameters. The differences between 1D BRNN models trained with real and synthetic cases in 18 real test cases were significant for all three predicted hemodynamic parameters: PD (25.18 ± 20.95 mmHg vs. 9.61 with [1.39–39.06], Wilcoxon test, *p* = 0.006), WSS_max_ (33.92 ± 20.71 Pa vs. 38.59 with [23.61–78.52] Pa, Wilcoxon test, *p* < 0.001), and V_max_ (3.13 ± 1.47 m/s vs. 2.92 with [2.04–4.81] m/s, Wilcoxon test, *p* < 0.001).

### 3.3 Impact of an aortic arch shape on ANN training: gothic vs. non-gothic vs. mixed cohorts

Statistical analysis of the predictions from the three models *versus* CFD results for all 30 test cases revealed significant differences in mean error. The gothic model had a significantly higher mean error of −8.41 ± 9.46 mmHg (paired Student’s t-test, *p* < 0.001 for both tests) compared to the non-gothic and mixed models, which had −2.76 ± 8.80 mmHg and −4.27 ± 10.10 mmHg, respectively. However, the differences in errors between the non-gothic and mixed models were non-significant (paired Student’s t-test, *p* = 0.092).

**FIGURE 8:**
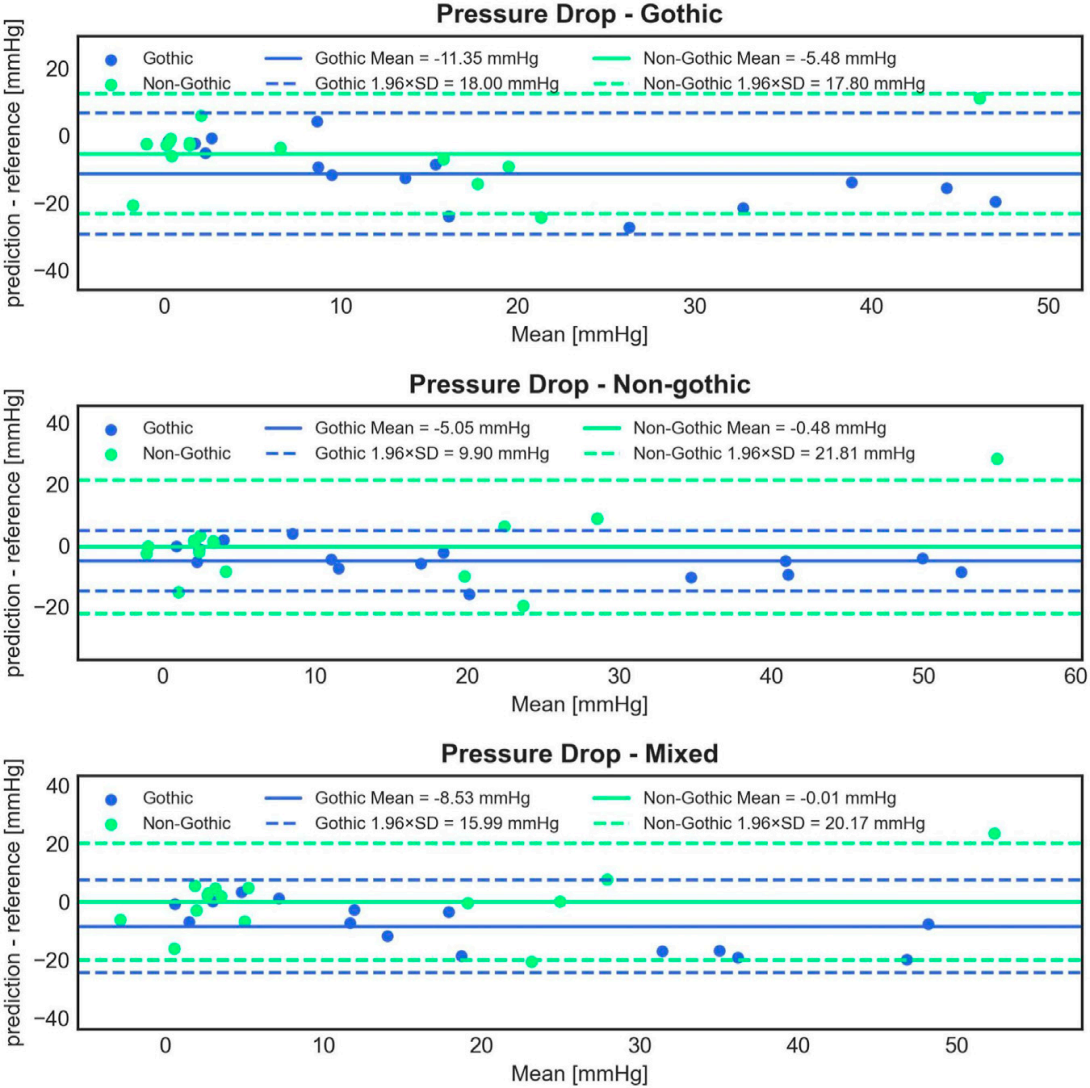
Bland-Altman plots comparing the accuracy of 1D BRNN PD predictions on 15 gothic and 15 non-gothic test cases for the following experiments: trained only on gothic cases (1st row) vs. only on non-gothic cases (2nd row) vs. on mixed cases (3rd row).

Figure 8 illustrates Bland-Altman plots for all three trained models, separated for gothic and non-gothic test cases. The model trained with only gothic cases had a higher mean PD prediction error for gothic test cases compared to non-gothic cases (−11.35 ± 9.18 mmHg vs. −5.48 ± 9.08 mmHg). However, this difference was not significant (Student’s t-test, *p* = 0.089). Similar results were found for the model trained with non-gothic cases (−5.05 ± 5.05 mmHg vs. −0.48 ± 11.13 mmHg) as well as for the mixed model (−8.53 ± 8.16 mmHg vs. −0.01 ± 10.59 mmHg). In the non-gothic model, the differences were not statistically significant (paired Student’s t-test, *p* = 0.158), whereas, for the mixed model, the predictions for non-gothic test cases were significantly more accurate (paired Student’s t-test, *p* = 0.018).

Interestingly, the model trained with non-gothic cases significantly outperformed the gothic model in predicting gothic test cases (paired Student’s t-test, *p* < 0.001) and the mixed model (paired Student’s t-test, *p* = 0.01). For non-gothic test cases, the performance of the gothic model was significantly less accurate compared to the non-gothic (paired Student’s t-test, *p* = 0.013) and mixed models (paired Student’s t-test, *p* = 0.003), with no significant difference between non-gothic and mixed models (paired Student’s t-test, *p* = 0.668).

### 3.4 Impact of an architecture on ANN training: 1D vs. 3D

**FIGURE 9:**
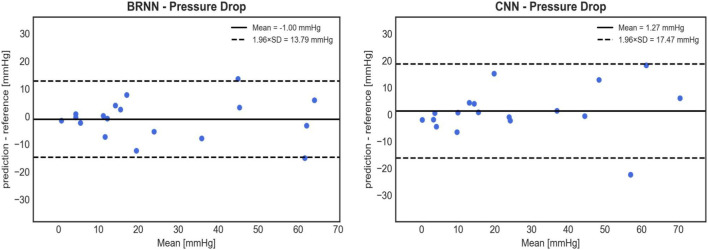
Bland-Altman plots comparing the accuracy of PD predictions on 18 test cases between the 1D BRNN (left) and 3D CNN (right). CNN’s cross-sectional values were averaged to get 1D pressure values along the centerline.

**FIGURE 10:**
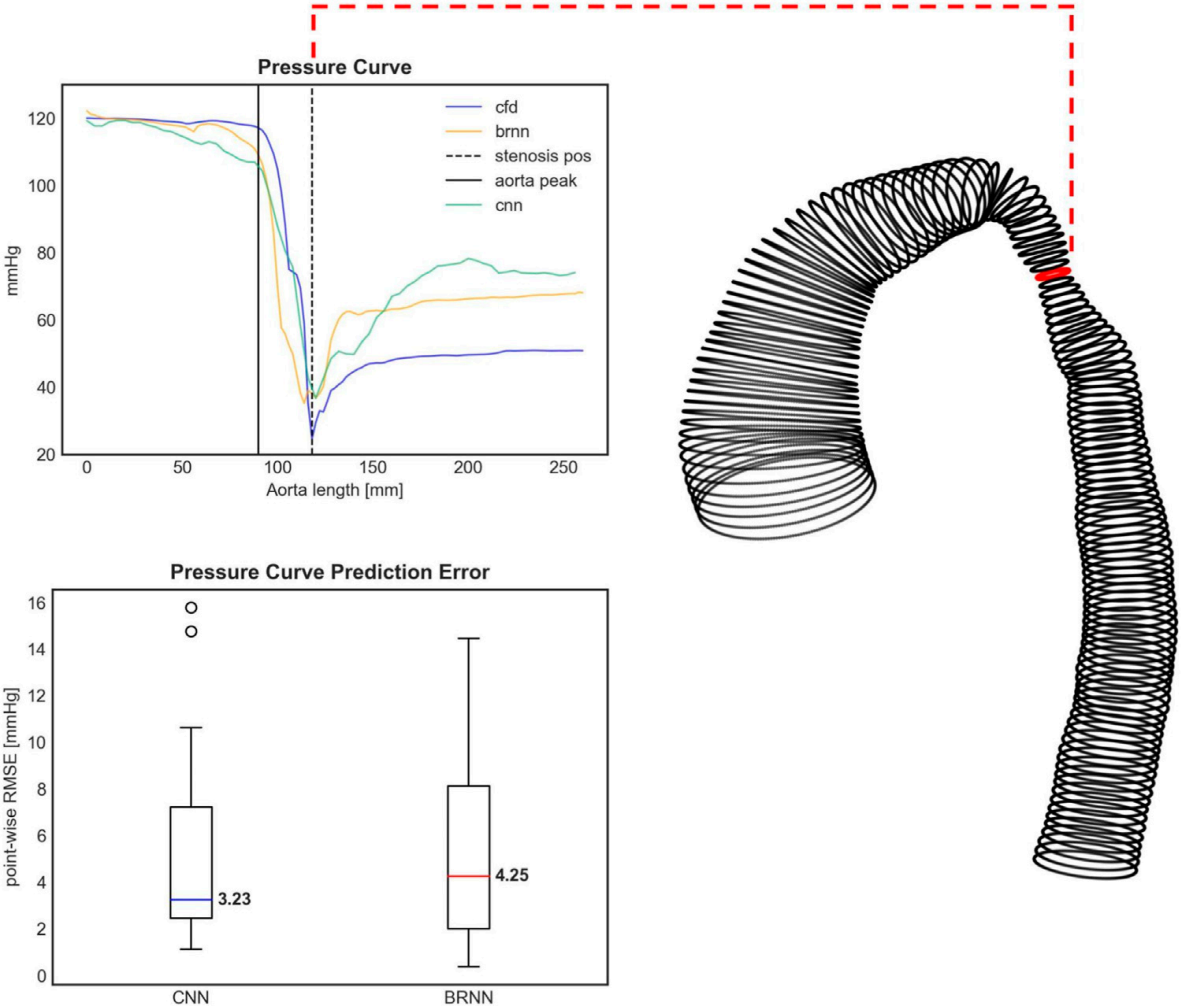
Upper figure: The pressure curve for an exemplary test case with the highest pressure drop (PD) prediction error. Both the 3D CNN and 1D BRNN overestimate the pressure recovery after the stenosis, thus affecting the PD prediction. The PD occurs right at the aorta’s narrowing (dashed black line). The pressure course remains relatively steady at the aortic arch (black solid line). The shape of this specific case can be seen on the right side with the red circle marking the stenosis site with the lowest diameter. Bottom figure: Boxplots comparing the error of pressure curve predictions between the 1D BRNN and 3D CNN by computing point-wise RMSE for all 18 test cases.

Based on the analysis of the mean and standard deviation of the prediction errors (Figure 9), the 3D CNN exhibits a slightly higher error (1.27 ± 8.91 mmHg) compared to the 1D BRNN (−1.00 ± 7.04 mmHg) in predicting PD. However, statistical analysis found no significant differences when comparing PD values predicted by the 3D CNN to those calculated by the CFD (26.19 ± 23.29 mmHg vs. 14.79 with [9.96–40.79] mmHg, Wilcoxon test, *p* = 0.711) as well as between PD values predicted by 3D CNN and those predicted by the 1D BRNN (26.19 ± 23.29 mmHg vs. 16.52 with [7.17–48.15] mmHg, Wilcoxon test, *p* = 0.528). The higher standard deviation of the 3D CNN error can be primarily attributed to one outlier (Figure 10), where both the 1D BRNN and 3D CNN overestimated the pressure recovery after the stenosis.

Similar results were obtained when examining the point-wise RMSE of pressure curves (Figure 10). The 3D CNN exhibited a lower median RMSE (3.23 mmHg) compared to the 1D BRNN (4.25 mmHg), however, this difference was not significant (paired Wilcoxon test, *p* = 0.879). The IQR was also narrower for 3D CNN. However, it should be noted that there were two outliers in the case of the 3D CNN, which did not appear in the results of the 1D BRNN. Overall, it can be concluded that the 1D BRNN demonstrated slightly better accuracy in predicting PD, while the 3D model marginally outperformed the 1D model in terms of pressure profile accuracy.

Finally, a sensitivity and specificity analysis were conducted on test cases using a PD threshold of 20 mmHg, following current guidelines that recommend intervention if the peak-to-peak coarctation gradient exceeds 20 mmHg at rest (Mercuri et al., 2020). The CFD results served as the ground truth and were compared with the 1D BRNN, which performed slightly better than the 3D CNN in predicting PD (Figure 9). The results of this analysis are as follows: 7 cases were true positive, 9 cases were true negative, 1 case was false positive, and 1 case was false negative. This yields a sensitivity of 87.5% and a specificity of 90%.

## 4 Discussion

The selection of BRNN for predicting hemodynamics along the 1D centerline of the aorta appears to be a reasonable choice, considering that centerline points exhibit sequential dependencies. BRNNs are well-suited for modeling such dependencies in both forward and backward directions. Furthermore, research has suggested that CNNs trained on small data with a saturated activation function like Leaky ReLU perform better than those trained with the standard ReLU, which has a zero-slope part for negative values (Xu et al., 2015).

Both our models 1D BRNN and 3D CNN were trained with unscaled data, with the exception of the output 3D CNN data, which was scaled by standard deviation. Surprisingly, the models trained on non-scaled data performed the best, which is not aligned with the common machine learning practice of scaling the training data. In our case, standardization and normalization of data resulted in overfitting of the 1D BRNN, where the training loss continued to improve while the validation loss converged or even increased. Similarly, although scaling did not result in overfitting for the 3D CNN, this model also performed better with unscaled input data. This raises the question of why the models trained with unscaled input data show better accuracy. One possible explanation is that certain input features with smaller scales (e.g., centerline coordinates) might be less predictive, implying they contain less information about the hemodynamic outputs obtained from the ANN. Essentially, because the less predictive features have smaller scales and the more predictive features have larger scales, these more predictive features might naturally have a greater impact on the output. This is the exact scenario we usually want to avoid in machine learning, where we aim to prevent the scale of input and output data from introducing biases. However, in our case, these biases might have actually been beneficial. It is important to emphasize that more predictive features in the ANN model do not necessarily align with the physical reality of the most predictive factors for hemodynamic calculation.

To optimize the weights, the Adam optimizer was used, which yielded the best results in cross-validation experiments. This is not surprising, as it has already been shown before (Kingma and Ba, 2014) that Adam can be a better alternative to other optimizers such as stochastic gradient descent (SGD) and root mean squared propagation (RMSProp).

The performance of the 1D BRNN in predicting PD, with a standard deviation error of 7.03 mmHg, suggests that this approach has the potential to be used in clinical practice for the diagnosis of CoA patients. The high sensitivity and specificity, both around 90%, further indicate that the model’s error would not be a limiting factor for clinical diagnosis. Moreover, the model consistently and accurately predicts the position of the stenosis (where the static relative pressure drops) in all CoA test cases.

To improve PD prediction reliability, the introduction of confidence intervals could be considered. If the predicted PD value falls within the range of one standard deviation error (7.03 mmHg), additional investigation would be recommended. To a certain extent, adhering to confidence intervals already exists in clinical practices, where doctors, using cardiac catheterization for determining peak-to-peak pressure gradient, acknowledge an error margin of around 5 mmHg (Yevtushenko et al., 2022).

Furthermore, the 1D BRNN showed consistent errors in the prediction of PD, WSS_max_, and V_max_ across a wide intensity spectrum, as evidenced by the Bland-Altman plots (Figure 5). The absence of outliers as hemodynamic values increase indicates that the model has the potential to generalize well to a broader patient population.

It turns out that training the CNN with 3D aorta geometry and pressure distribution does not result in improved predictions of PD, although the pressure curves showed a slight improvement in accuracy. However, this approach has its drawbacks, including increased spacing between cross-sections (4 mm instead of 2 mm) and the limitation of predicting only static pressure. This decision was made to maintain a reasonable batch size for training, which was 8 in our case. If additional output features such as a 3D velocity field are added or the spacing between cross-sections is reduced, the batch size would need to be decreased to fit into GPU RAM. It is preferable to have a reasonably high batch size (with 32 being a good rule of thumb), as lower batch sizes can lead to less accurate predictions (Radiuk, 2017). It is worth noting that a 3D model could be developed, where the first step involves a rough calculation of 3D velocity fields inside the aorta using CFD-calculated velocity fields for training. The information from this step could then be used in the second step for predicting the pressure fields. In other words, we could train the model to predict velocity fields instead of pressure and still be able to predict pressure.

One potential solution to address GPU RAM limitation could be reducing the dimensions of cross-sectional planes. For instance, resizing the 3D input and output arrays from (48, 48, 80) to (24, 24, 160) would reduce the overall array size by a factor of 2 while also reducing the spacing to 2 mm. However, it is found that this resizing approach results in lower accuracy of pressure profile prediction, with a median error of 4.34 mmHg, which is 34% higher compared to the 3.23 mmHg obtained by the non-resized solution. Despite experimenting with various shapes, the final shape chosen for the input and output arrays was (48, 48, 80), as it yielded the best results.

The lower batch size (8 for the 3D CNN) compared to the 1D BRNN (50) due to GPU RAM limitations highlights the challenge posed by the higher-order complexity of transitioning from 1D to 3D. Additionally, there is a presence of redundant information among input features, such as radius, gradient of radius, flow, and velocity through cross-sections, which are originally 1D features but need to be presented as 3D arrays (with constant values on cross-sections) to comply with the 3D CNN architecture requirements. Although CNNs are commonly used in state-of-the-art machine learning practices, especially for classifying and segmenting imaging data, their ability to model sequential dependencies, similar to RNNs, is questionable.

In conclusion, a better-suited machine learning architecture, combining the strengths of both, CNN (for convolutional layers) and BRNN (for modeling sequential dependencies), while reducing the information redundancy and retaining 3D geometry could be identified.

The experiments with different training data were conducted to investigate whether there are any significant alterations in performance, which can provide insights into the underlying distribution of different datasets. Interestingly, it is observed that the 1D BRNN performs better when trained solely on real cases compared to when trained exclusively with synthetic cases. This difference in performance suggests that there may be variations in the data distributions between the two cohorts. It is possible that the synthetic cohort does not fully represent the entire distribution of the clinical cohort, leading to the underperformance of the model trained on synthetic data.

The model trained on the clinical cohort is expected to generalize better on unseen data if its training data better reflects the true distribution of the population. Although the distribution of flow hemodynamics (PD, WSS_max_, and V_max_) is similar between the real and synthetic cohorts, it has been observed before (12) that the stenosis degree and stenosis position distributions do not match well between the clinical and synthetic cohorts, which might suggest potential flaws in the construction of the synthetic cohort.

Interestingly enough, there were no significant improvements in the performance of the 1D BRNN when it was trained with gothic, non-gothic, or mixed (50% gothic and 50% non-gothic) synthetic cases. The results of this experiment suggest that, despite some statistically significant differences between the models, they could not effectively capture the distinctions between the gothic and non-gothic cases. The outcome was somewhat unexpected, as you might assume that the model would perform better for the cases it was trained on.

Moreover, these results could also be attributed to the fact that all the training cases come from the synthetic cohort, which was found not to be equivalent to the clinical cohort in terms of producing comparable model accuracy. What is particularly interesting is that the gothic model performed significantly worse than the non-gothic model for both gothic and non-gothic test cases, alluding that adding another kind of the flow resistance (i.e., aortic arch) might have confused the ANN model. In other words, the model was not able to differentiate the pressure drop caused by the aortic curvature or the narrowing. Also, it seems that the model trained with non-gothic cases is also able to learn to predict the pressure drop in gothic cases. This finding, that even the model trained with no gothic cases can predict PD reasonably well for gothic cases, is important since gothic cases are seldom found in a real cohort and it is challenging to collect a large number of clinical gothic cases to train the ANN.

However, there exists a reasonable doubt about the validity of the results from the gothic experiment. Firstly, looking at Figure 8, some cases can be distinctly identified where the mean value between the predicted and reference values is negative. It is very much possible that the model predicts a higher pressure at the outlet than at the inlet, resulting in a negative pressure drop. This situation may occur when the pressure remains relatively constant throughout the aorta, which is typical for healthy subjects without stenosis. This showcases one of the limitations of the ML models, as they lack awareness of the physical reality present in the given problem they attempt to solve. One approach to tackling this limitation is to “teach” the model about what is physically plausible by introducing penalty terms to the cost function. For example, during the training process, if the model predicts a negative pressure drop, a penalty term could be added to the cost function, aiming to avoid this outcome.

Secondly, some cases show extremely high errors, reaching magnitudes of 100%. This could stem from training exclusively on synthetic cases and having a relatively small training sample size. Our findings, illustrated in Figure 7, indicate that the models trained only on synthetic cases perform significantly worse than those trained only on real cases. We used in this experiment gothic cases within the synthetic cohort, as there are not many among real cases.

Further, the hemodynamic distributions among all three groups are similar. However, upon closer examination, it is found that gothic cases within the clinical cohort exhibit more severe hemodynamics compared to non-gothic real cases. The same observation does not hold true for the synthetic cohort, suggesting that the synthetic cases may not accurately capture the impact of the gothic arch on hemodynamics.

Considering these findings, further experimentation with different methods for generating synthetic cases should be conducted in the future. Improving the synthetic cohort could potentially enhance the performance of the ANN even further.

Finally, we must note that the current study is limited to the quasi-steady CFD model employed, which only calculated peak systolic flow conditions in a pulsatile flow. Unsteady flow simulations of the aortic flow are associated with high computational costs, which are, based on our experience, approximately 10-fold compared with a peak-systolic flow simulation. This is because at least two heart cycles have to be simulated to achieve a time-independent solution and because usual time steps between 0.0004 s and 0.005 s (Qin et al., 2023) used for aortic flow simulations result in approximately a few hundred or thousands of time steps to be simulated. Unsteady flow simulations are necessary to assess velocity and pressure fields accurately, especially during high flow acceleration and deceleration phases. However, during peak systole, which is the single time point used for the CoA pressure gradient assessment according to the clinical guideline, the impact of flow unsteadiness is considered to be negligible. This assumption for the CFD model used in our study is confirmed by a set of clinical validation studies against *in vivo* catheter-measured pressure gradients vs. 4D PC MRI measurements (Mirzaee et al., 2017; Bouaou et al., 2019; Goubergrits et al., 2019; Shi et al., 2019).

## 5 Conclusion

This study showcases the potential of ML methods to replace CFD, effectively mitigating computational costs and facilitating their integration into clinical practice. The inclusion of 3D geometric and pressure information does not boost ANN accuracy in predicting PD. This leads to the conclusion that condensing geometric and flow hemodynamics information into a 1D representation along the aorta centerline is a reasonable simplifying approach. The introduction of a synthetic cohort to augment geometric and hemodynamic variability does not yield an improvement in ANN performance, but it does demonstrate the suitability of synthetic data for training ML models. Consequently, future studies should focus on improving data augmentation techniques to potentially acquire better ML models. Finally, ML models do not seem to identify the variations between cases that have different shapes of the aortic arch. Additionally, exploring alternative architecture could also lead to further improvements.

## Data Availability

Publicly available datasets were analyzed in this study. This data can be found here: https://www.morphosource.org/projects/0000C1064 DOI 10.6084/m9.figshare.13568234.
